# A scoping study of practitioner training in alcohol brief interventions as described and delivered in randomised controlled trials

**DOI:** 10.1186/1940-0640-10-S2-O38

**Published:** 2015-09-24

**Authors:** Niamh Fitzgerald, Kathryn Angus, Jonathan Bryce, Linda Bauld

**Affiliations:** 1 Institute for Social Marketing, UK Centre for Tobacco and Alcohol Studies, School of Health Sciences, University of Stirling, Stirling, UK

## Background

Good evidence for the efficacy of alcohol brief interventions (ABIs) delivered in primary care [1] has been insufficient to drive routine implementation [2] and may not translate to normal clinical settings, possibly due to a loss of fidelity [3]. Implementation efforts, and research trials, rely on practitioners being adequately prepared to deliver ABIs consistently with evidence or protocols (amongst other factors). Relatively little research attention has been paid to establishing optimal duration, content, methods, style, deliverer or format of training and the importance of heterogeneity in training provision in affecting trial outcomes is currently unknown.

This study aimed to ascertain how published trials of ABIs describe the training and related support provided to practitioners, what additional relevant information can be obtained from trial authors and how current frameworks or taxonomies can inform how best to analyse and describe training content and design.

## Materials and methods

A systematic search identified published ABI trials involving frontline healthcare workers. Data on how training is reported in papers and supplementary materials was extracted so as to facilitate analysis of how clearly the design, duration, method and topics covered in training are described and reported. Corresponding authors were contacted to request further information on training content and design. Existing frameworks which might facilitate systematic description of ABI training will be critically appraised to assess usefulness for routine use in reporting.

## Results

780 records were identified from the Cochrane Drug and Alcohol group specialised register and relevant Cochrane systematic reviews. After de-duping and screening, 221 full text articles were being assessed for eligibility and data extracted on training and support provided to practitioners in eligible reviews.

## Conclusions

Preliminary findings suggest that the quality of descriptions of training, as well as the duration and intensity of training and support provided to practitioners are highly variable and that in future standardised reporting would facilitate further analysis.
